# Neural Mechanisms Underlying the Auditory Looming Bias

**DOI:** 10.1080/25742442.2021.1977582

**Published:** 2021-09-20

**Authors:** Karolina Ignatiadis, Diane Baier, Brigitta Tóth, Robert Baumgartner

**Affiliations:** a Acoustics Research Institute, Austrian Academy of Sciences, Vienna, Austria; b Center for Natural Sciences, Institute of Cognitive Neuroscience and Psychology, Budapest, Hungary; c Faculty of Education and Psychology, Eotvos Lorand University, Budapest, Hungary

**Keywords:** looming, electroencephalography, connectivity

## Abstract

Our auditory system constantly keeps track of our environment, informing us about our surroundings and warning us of potential threats. The auditory looming bias is an early perceptual phenomenon, reflecting higher alertness of listeners to approaching auditory objects, rather than to receding ones. Experimentally, this sensation has been elicited by using both intensity-varying stimuli, as well as spectrally varying stimuli with constant intensity. Following the intensity-based approach, recent research delving into the cortical mechanisms underlying the looming bias argues for top-down signaling from the prefrontal cortex to the auditory cortex in order to prioritize approaching over receding sonic motion. We here test the generalizability of that finding to spectrally induced looms by re-analyzing previously published data. Our results indicate the promoted top-down projection but at time points slightly preceding the motion onset and thus considered to reflect a bias driven by anticipation. At time points following the motion onset, our findings show a bottom-up bias along the dorsal auditory pathway directed toward the prefrontal cortex.

## Introduction

Likely for the sake of hazard prevention, looming sounds are perceived as more salient to listeners than receding ones, in a rather fast, probably automatic evaluation process; this effect is termed the "auditory looming bias". As such it manifests in various forms, across species and ages: rodent studies demonstrate higher cortical activity (Deneux, Kempf, Daret, Ponsot, & Bathellier, 2016) while primates show increased attention (Ghazanfar, Neuhoff, & Logothetis, 2002) toward looming signals. In humans, evidence of an automatic process has been found through stimuli imperceptibly rising in intensity, which lead to increased visual cortex activation (Romei, Murray, Cappe, & Thut, 2009). The auditory looming bias has shown its presence in loudness changes (Neuhoff, 1998; Seifritz et al., 2002), through alertness to perceived speed (Neuhoff, 2016) and relative distance (Neuhoff, Planisek, & Seifritz, 2009) of objects, as well as overall faster reaction times (Bidelman & Myers, 2020). In line with an evolutionary explanation (Neuhoff, 2001), the looming bias emerges stronger in physically weak or vulnerable people (Neuhoff, Long, & Worthington, 2012), as well as under mentally challenging conditions (increased cognitive load; McGuire, Gillath, & Vitevitch, 2016). Interestingly, behavioral indications for prioritizing auditory looms were demonstrated even in newborns as well as infants of 4 months of age, who most presumably are not yet aware of the hazardous nature of approaching objects (Morrongiello, Hewitt, & Gotowiec, 1991; Orioli, Bremner, & Farroni, 2018).

Although the precise cortical mechanisms behind this looming bias are not yet clearly understood, parts of the hypothesized networks have been extensively studied. Inferior parietal areas of the cortex (inferior parietal lobule; IPL) have been found crucial for spatial hearing in general (Majdak, Baumgartner, & Jenny, 2020; van der Heijden, Rauschecker, de Gelder, & Formisano, 2019) and sound motion processing in particular (Warren, Zielinski, Green, Rauschecker, & Griffiths, 2002). Functional magnetic resonance imaging (fMRI) investigations have specifically highlighted the involvement of the amygdala, temporal plane, superior temporal sulcus and intraparietal sulcus in the auditory looming circuit as well prefrontal cortex (PFC), primary auditory cortex (PAC) and IPL in the preferential processing of looming sounds (Bach et al., 2008; Seifritz et al., 2002).

As apparent from those previous findings, there are distributed networks involved in the processing of looming sounds but studies based on fMRI have not revealed the timing of underlying processes because of limited temporal resolution. The high temporal resolution of magneto- or electroencephalography (M/EEG) allows for unraveling such network dynamics through connectivity analyses. Specifically, functional connectivity quantifies how different brain areas communicate with each other to perform a given task (Stam & van Straaten, 2012). Recent connectivity analyses argue that top-down directional causal influence from PFC to PAC enhances processing of looming versus receding sounds (Bidelman & Myers, 2020).

That study as well as almost all previous ones examined auditory looming bias using intensity-based distance simulations (Bach, Furl, Barnes, & Dolan, 2015; Bach, Neuhoff, Perrig, & Seifritz, 2009; Bach et al., 2008; Bidelman & Myers, 2020; Deneux et al., 2016; Ghazanfar et al., 2002; Morrongiello et al., 1991; Neuhoff, 1998, 2016; Neuhoff et al., 2009; Orioli et al., 2018; Romei et al., 2009; Seifritz et al., 2002). Intensity-based stimuli are constructed by rising, falling or constant intensity. As intuitively understood, when a sound source approaches, its intensity increases. Varying the intensity of a presented sound is, therefore, a simple and effective way to simulate motion along the distance dimension. Yet when examining the neural substrate of auditory mechanisms, increases in sound intensity are potential confounders of the observed changes: listeners' judgments of auditory sweep duration as well as response sensitivity have been found to be influenced by stimulus intensity level (Herrmann, Augereau, & Johnsrude, 2020; Teghtsoonian, Teghtsoonian, & Canévet, 2005).

**Figure 1:**
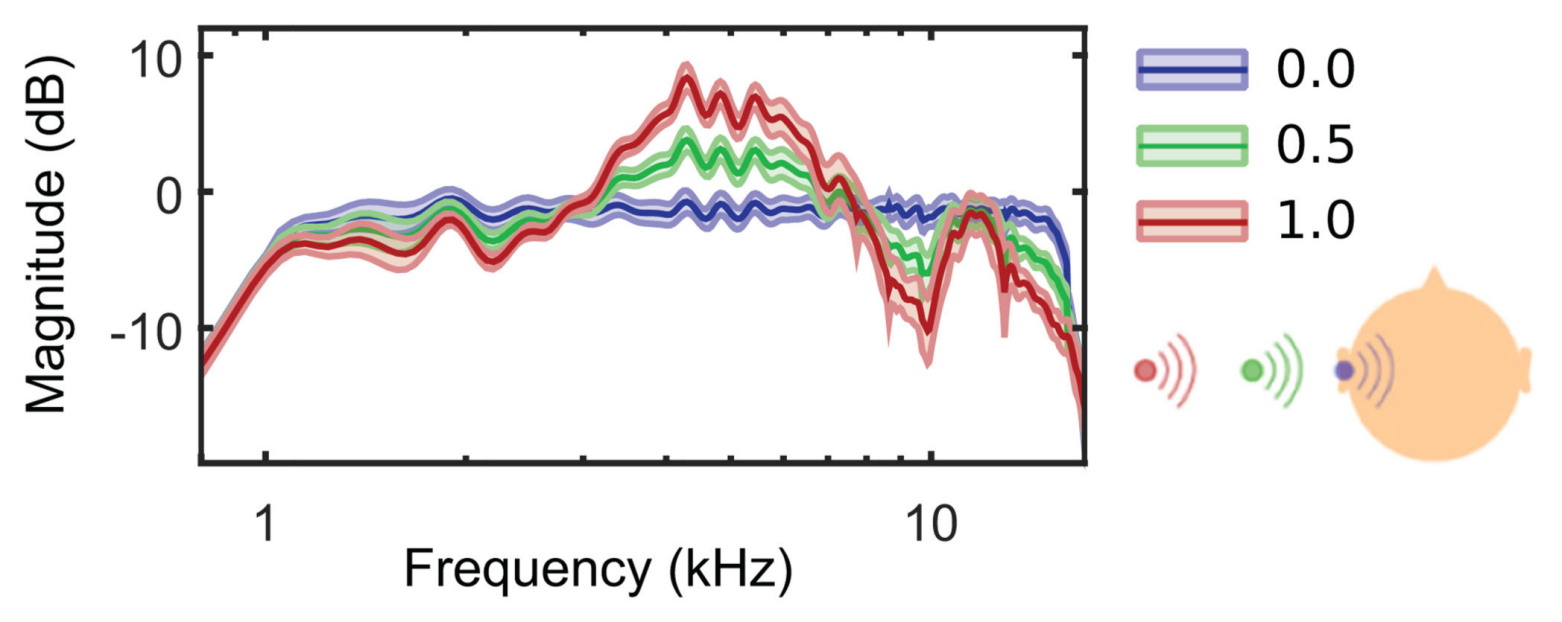
Magnitude responses of listener-specific stimuli as a result of spectral contrast factor C manipulation. Different factor values correspond to different simulated positions in space: C = 1 for an external position (red), C = 0 for a perceived location inside the listener's head (blue), C = 0.5 an intermediate of the two (green). [Adapted from Baumgartner et al. (2017)].

To avoid this confound, a previous work has implemented stimuli that maintained their overall intensity while eliciting a looming percept by manipulating spatial spectral cues of the sound instead (Baumgartner et al., 2017). Spectral cues are naturally induced via acoustic filtering of incoming sounds by features of the human morphology. Specifically, the shape of the human pinna causes those cues to be more pronounced in the high frequencies. They are essential for the externalized perception of sound sources (Best, Baumgartner, Lavandier, Majdak, & Kopčo, 2020) and thus, spectral cue manipulation can create the percept of a sound source moving along the distance dimension (Figure 1). Using such stimuli allowed to demonstrate significantly increased EEG activity within the 120–200 ms interval after looming onset.

In the present work, we performed a connectivity re-analysis on those previously collected EEG data (Baumgartner, 2017), aiming to identify auditory cognitive subsystems that underlie the auditory looming bias. Our aim was to investigate the potential modulation between PFC and the primary auditory cortex (PAC), following a looming stimulus presentation. We specifically looked into whether the top-down connectivity, as suggested by recent research (Bidelman & Myers, 2020), is generalizable across different auditory spatial cues (intensity or spectral shape changes). In addition, we explored the potential involvement of other cortical regions but in particular sensory association cortices (parietal areas) in promoting the looming bias.

## Materials and Methods

### Subjects

For the present study, we used the publicly available data (Baumgartner, 2017) from a former study (Baumgartner et al., 2017). The data set contains EEG recordings and behavioral data from a total of 15 listeners (age 20–29 y, M = 24, SD = 3.7; 10 females). As the available sample size is rather small for connectivity analyses, we ran post-hoc power analyses based on our effect sizes and an alpha of 0.05 for all findings and included the values in all reported results. Audiometric thresholds for frequencies in the range 0.5–8 kHz revealed no hearing loss greater than 20 dB relative to the normal-hearing population for any of the participants.

### Stimuli

As described in full detail in Baumgartner et al. (2017), stimuli consisted of two consecutively presented 600 ms long Gaussian white noises with a bandwidth limited to the range 1–16 kHz. In order to manipulate the perception of the sound source's spatial position on the horizontal plane the stimuli were filtered with the individual listener-specific head-related transfer functions (HRTFs). The spectral contrast factor C was varied among three values, C = {0, 0.5, 1}, while the overall sound intensity remained unchanged. C = 1 represents the original spectral profile (measured 1.5 m from the listener's head), while C = 0 corresponds to a flat spectral profile creating an internal percept within the listener's head. C = 0.5 simulates a position between the aforementioned two (Figure 1). Looming trials consisted of switches from C = 1 to C = 0.5 (half step; predictable), from C = 0.5 to C = 0 (half step; unpredictable) and from C = 1 to C = 0 (full step; predictable), while receding trials switched from C = 0 to C = 0.5 (half step; predictable), from C = 0.5 to C = 1 (half step; unpredictable) and from C = 0 to C = 1 (full step; predictable). All stimuli were presented binaurally via tubephones (ER-2, Etymotic Research) without an inter-stimulus interval (ISI) between the spectral changes, thus eliciting the perception of motion of a single sound object.

### Procedure

**Figure 2:**
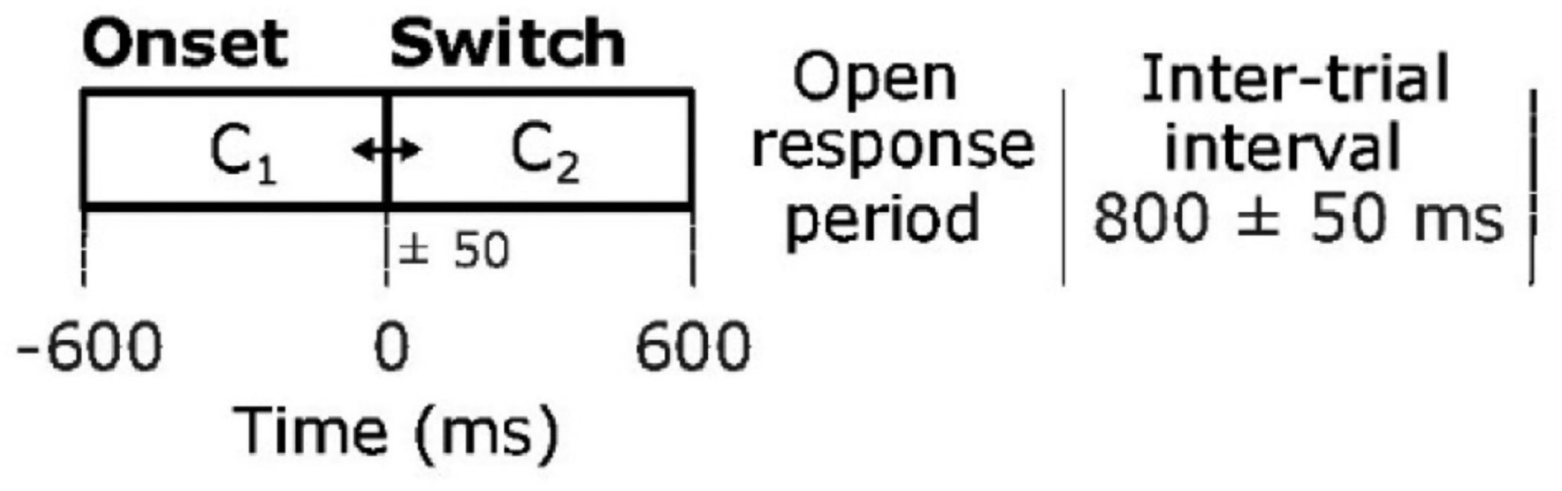
The spectral contrast factor C was manipulated to create looming and receding sounds. Noise filtered with factor C_1_ was cross-faded to C_2_ with a temporal jitter of ±50 ms. Listeners were asked to report whether the sound was approaching, receding or static during the open response period. [Adapted from Baumgartner et al. (2017)].

The experimental paradigm consisted of a motion direction discrimination task (Figure 2). By varying factor C, looming (C_1_ > C_2_), receding (C_1_ < C_2_) or static C_1_ = C_2_ stimuli were created among external (C = 1), intermediate (C = 0.5) and internal (C = 0) positions. Listeners were asked to judge the motion direction in all cases (looming, receding and static) while feedback was provided to them after short blocks of 21 trials, only regarding the hit rate of the static trials. The subjects listened to 840 randomized trials in total, of which 14% were static (no change in spectral contrast).

The event of interest for the present study is the onset of spectral contrast change, denoted as stimulus switch and time stamped at t = 0 in Figure 2. The overall duration of the sound comprises 1200 ms, followed by an open response period.

### Recordings & Analysis

EEG recordings were collected using a 32-channel scalp EEG with active electrodes (ActiveTwo BioSemi) at a sampling rate of 512 Hz. One vertical and two horizontal electrodes were placed around the eyes for measuring blinks and saccades. External reference electrodes were positioned at the mastoids and ear lobes.

The data was filtered (1–30 Hz) and re-referenced to the average. Independent component analysis (ICA) was applied for artifact removal. The data were then epoched in 3 second long segments, with a 1 second interval before sound switch and 2 after it, and a 200 ms baseline before each stimulus onset. As the available epoch length hindered analysis of very low frequencies, and in addition, high frequencies are not stable and reliable enough for phase dependent functional connectivity measures (van Diessen et al., 2015), we had to constrain analysis to the 1–30 Hz band. To estimate the volume conduction of cortical source activity, we numerically simulated a forward model using the boundary element method (BEM) as implemented in OpenMEEG (Gramfort, Papadopoulo, Olivi, & Clerc, 2010), with default MNI brain anatomy. To estimate source activity, we used the sLORETA inverse solution (Pascual-Marqui, 2002). In this algorithm, the voxels of the cortical volume are approximated by elementary dipoles. The current density normalization is done based on the noise covariance, calculated from the baseline interval, and the theoretical data covariance. In contrast to the actual data covariance, the theoretical covariance is extracted by the minimum norm solution of the forward model. The signals at 15.002 cortical surface points were reconstructed and then averaged for 62 cortical regions according to the parcellation scheme of Desikan-Killiany. Cortical regions were selected from each hemisphere for further analysis (region of interest – ROI), contralateral to the stimulus presentation site, as conscious hearing is processed contralateral to incoming sound direction (Nieuwenhuys, 1984). As motivated above we initially focused our analyses on the regions of PFC, PAC and IPL. As no connections between those regions indicated significant biases toward looms during the timing (120–200 ms) indicated by the previous event-related potential (ERP) study (Baumgartner et al., 2017), we subsequently performed a whole-brain analysis focused on that time range.

Regarding the connectivity analysis, we aimed for consistency with previous work (Bidelman & Myers, 2020) and applied the directed functional connectivity measure of phase transfer entropy (PTE; Lobier, Siebenhühner, Palva, and Palva 2014). PTE is an information theoretic measure that quantifies directed effects between time series. This measure operates based on the phase of the signal, thus being devoid of ambiguities the amplitude information can introduce.

Following a standard approach in time-frequency analysis, we applied the normalized PTE metric (dPTE) on the trial averages as well as on single trials. To obtain temporally resolved connectivity estimates, PTE was evaluated on a rectangular sliding window of 250 ms duration. Based on prior reports (Vicente, Wibral, Lindner, & Pipa, 2011) and our own experiences with using measures of PTE, this window length seemed to be a good trade-off between temporal resolution and the reliability of PTE estimates (Lobier et al., 2014). Using a shorter window length than that would not provide enough data to reliably estimate PTE. The step size was selected at 10 ms. Demonstrated in the abscissa of the corresponding plots is the center of said sliding time window (Figure 6(a,b,c)). Values for the looming bias were calculated as the difference between the looming and the receding conditions (collapsed over the different step sizes of spectral contrast manipulation if not stated otherwise) in directed connectivity between each pair of cortical regions considered. Inter-individual differences were out of the scope of this study because given the available data set it would not be possible to dissociate between differences in source localization accuracy based on template brain anatomy, and actual processing differences in the individuals' brain. We implemented the analysis pipeline in EEGLAB (Delorme & Makeig, 2004 for preprocessing) and Brainstorm (Tadel, Baillet, Mosher, Pantazis, & Leahy, 2011, for postprocessing).

Significance of the looming bias values was deemed based on cluster-based permutation tests of looming versus receding contrasts (Maris & Oostenveld, 2007): multi-subject time-series of dPTE differences between conditions (looming and receding) were permuted 2000 times, with a double-tailed t-statistic threshold of 0.95. Clusters were considered if ranging over at least 3 time windows (250 ± 15 ms). Effect sizes were calculated based on Cohen's d (Cohen, 1988).

## Results

### Behavioral Effects

**Figure 3:**
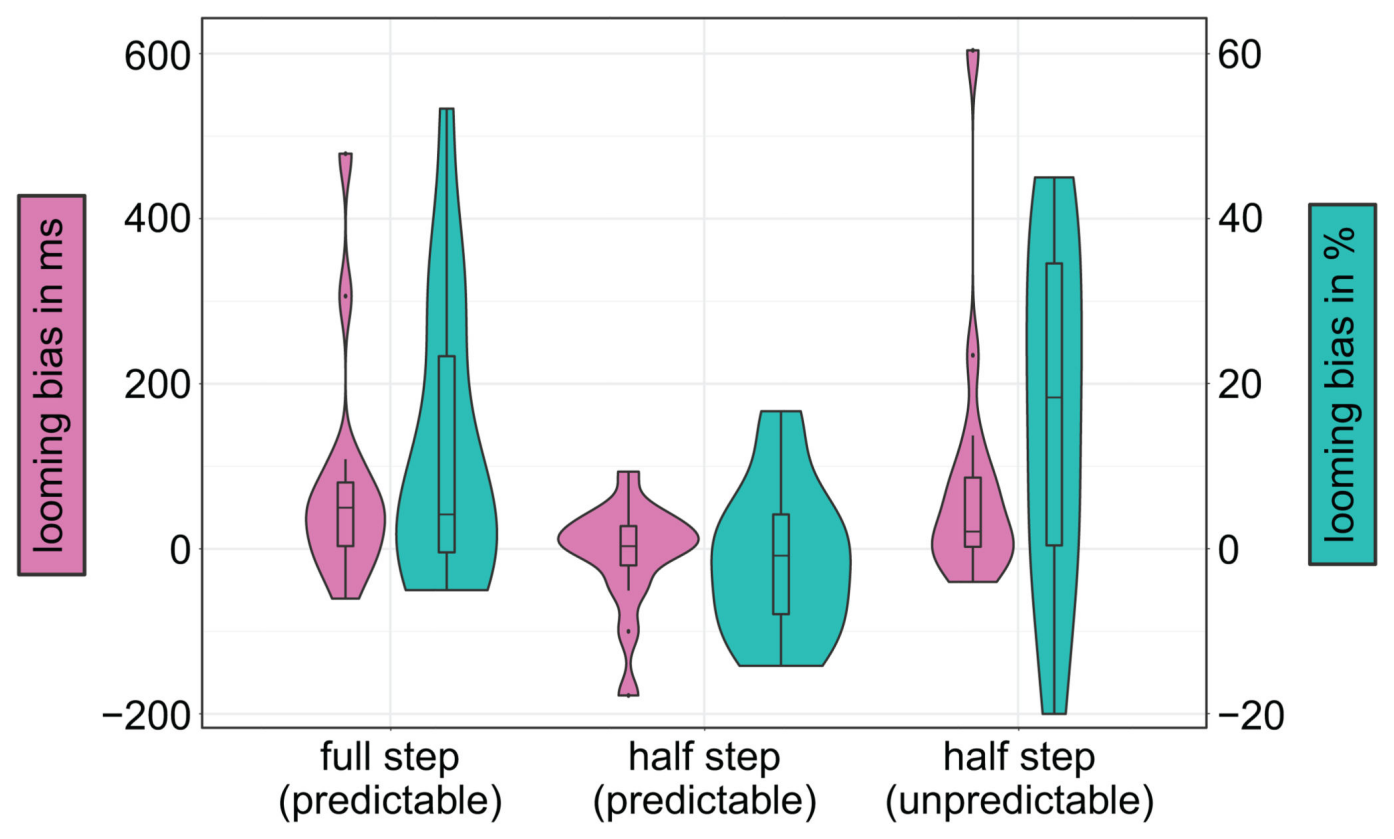
Violin and box plots depicting the median, interquartile range, and density of the looming bias of response times in ms (magenta, left x-axis) and accuracies in % (cyan, right x-axis) depending on the different step sizes of spectral contrast manipulation as well as predictability of the second stimulus on the y-axis.

In order to investigate the contribution of the different spectral-change conditions, full steps (from C = 0 to C = 1 or vice versa) and half steps (from C = 0 to C = 0.5 and C = 0.5 to C = 1, and vice versa), we re-analyzed the behavioral data from Baumgartner et al. (2017). Figure 3 shows the distribution of individual looming biases. For the accuracies, we ran a linear mixed-effect logistic regression model with spectral change (full steps; half steps) and direction of movement (looming; receding) as fixed effects and subject as random intercept and slope. The response times were analyzed with a general linear model with the same fixed and random effects. The results showed both significant main effects for direction and spectral change. For direction, subjects performed better (*z* = −2.34, *p* =.019) and faster (*t* = 2.97, *p* =.009) in looming (83.9%; 1.09 s) than receding trials (75.0%; 1.13 s). For spectral change, they performed better (*z* = −5.41, *p* <.001) and faster (*t* = 4.75, *p* <.001) for full (83.4%; 1.09 s) than half steps (77.4%; 1.12 s). Also, the interaction was significant (*z* = 4.24, *p* <.001): looming bias was larger for full than half steps (*t* = −3.01, *p* =.003).

### Source Activity

**Figure 4:**
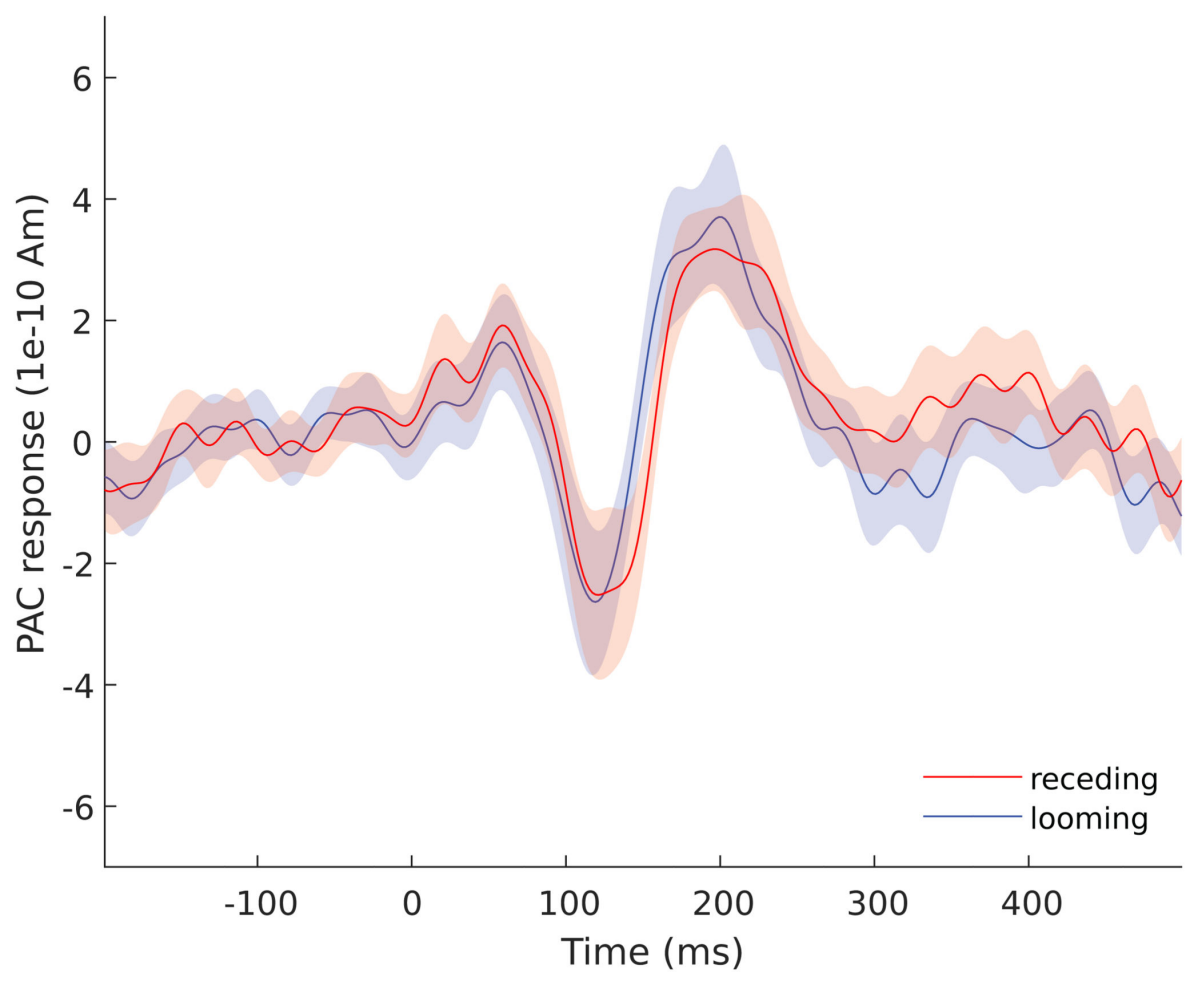
Group-average (N = 15) source strength responses in PAC elicited for trials with looming and receding stimuli. The PAC exhibits stereotypical auditory ERP dynamics (cluster-based permutation test).

We extracted the time series of our primary ROIs, that is, PAC, PFC, and IPL, for both the looming and the receding trials. The source activity obtained for PAC shows a stereotypical pattern of positive and negative deflections (Figure 4), indicating good localization accuracy despite the 32-channel EEG setup.

**Figure 5:**
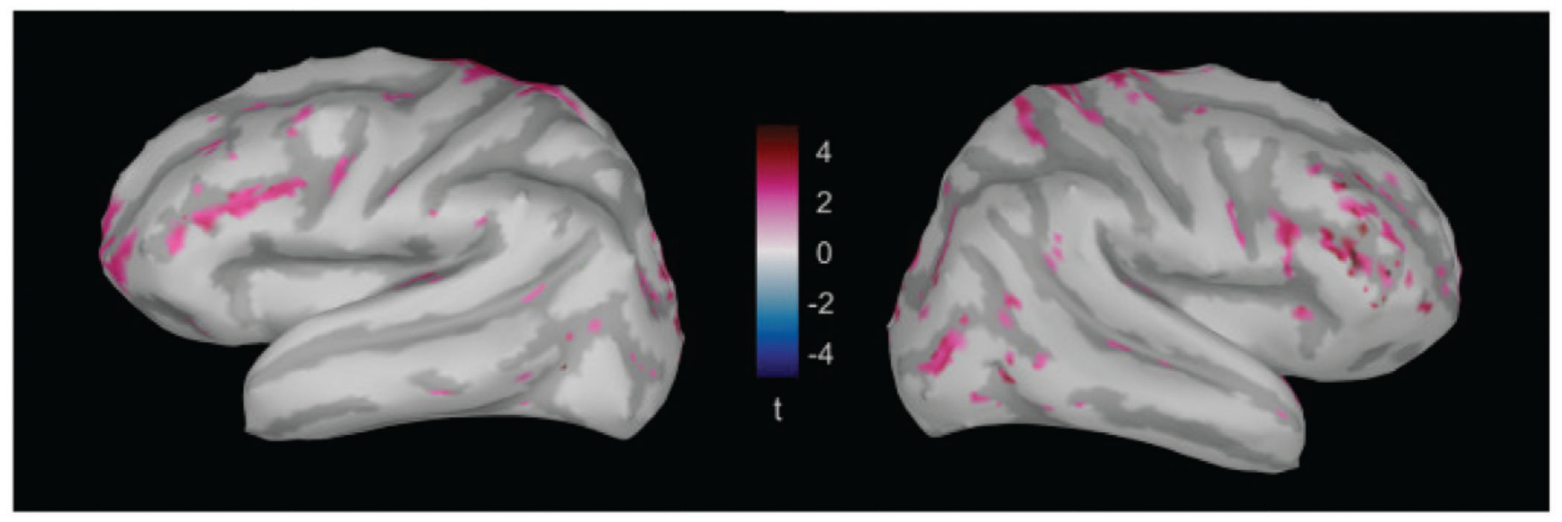
Brain maps highlighting the regions that show a significant emergence of the looming bias in the left and right hemisphere, averaged over the time window between 120 and 200 ms (Baumgartner et al., 2017). Differences are mostly localized in temporal, parietal and frontal regions.

To further explore other regions, we looked for significant differences in source activity over the whole brain while focusing on the time window between 120 and 200 ms, deemed vital in Baumgartner et al. (2017). Such differences are mostly localized in temporal, parietal and frontal regions (Figure 5). There, we found differences emerging in PAC on the temporal lobe, precentral gyrus (PreCG) on the parietal lobe, and postcentral gyrus (PostCG), superior frontal gyrus (SFG), pars triangularis (PTR), and pars opercularis (POP) on the frontal lobe.

### Connectivity

**Figure 6:**
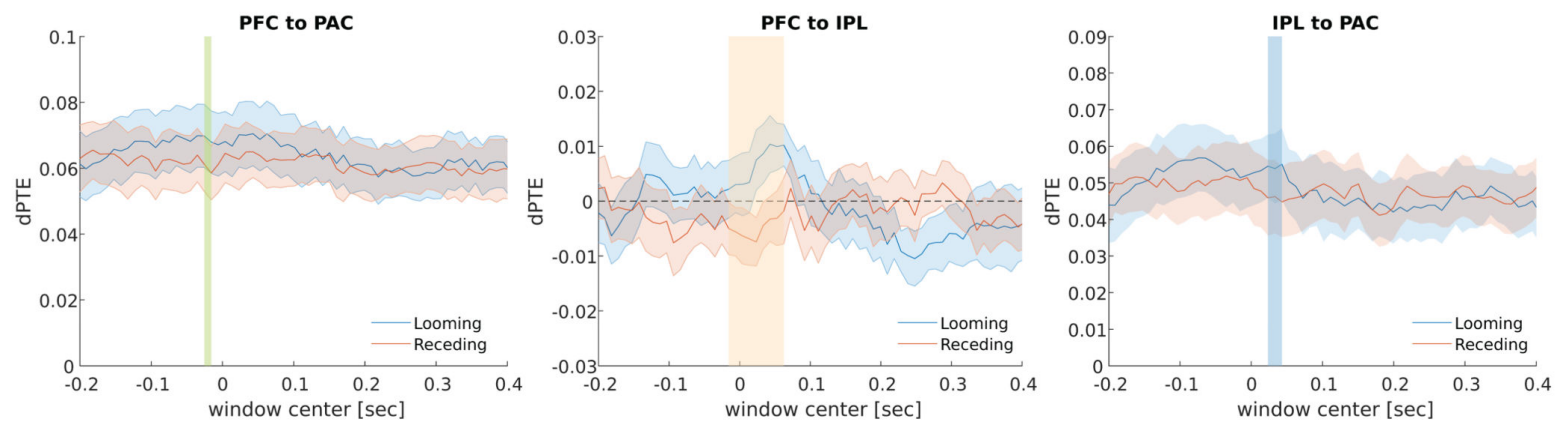
Group average connectivity values (dPTE, SEM, N = 15) between (a) PFC and PAC, (b) PFC and IPL and (c) IPL and PAC based on the individual single-trial source activities for looming and receding trials plotted against the center of the sliding time window (duration 250 ms). Significant occurrences of the looming bias are apparent at times around as well as before the stimulus change (vertical colored bars, *p* < 0.05; colors of the bars correspond to regions in fig. 7). Positive dPTE values represent connectivity in the targeted direction, indicated by the plot title, while negative ones denote the opposite direction.

We tested the dPTE connectivity between PFC and PAC based on both source signals averaged across trials as well as single-trial source signals. Across all time instances, connectivity was directed top-down from PFC to PAC, as indicated by positive dPTE values obtained from the single-trial analysis in Figure 6(a). A short time window slightly preceding the time of stimulus switch showed a rather weak but statistically significant connectivity increase in favor of looming sounds (gray shaded area; abscissa denotes the center of the sliding window; *d* = 0.55, *β* = 50.4%). This bias in PFC to PAC connectivity did not persist in individual analysis of predictable versus unpredictable conditions. Also, no significant instances of a looming bias were found in our analysis based on trial averages.

We further investigated the neural circuitry involving the IPL. With respect to PFC, the dPTE analysis of single-trial source activity revealed top-down directed connectivity from PFC to IPL in response to the looming stimulus (Figure 6(b)). Contrary to that, the receding stimuli elicited responses in the opposite direction (IPL to PFC). Instances of significant differences in the connections between the regions of IPL and PFC were found well during the time window of stimulus switch (*d* = 1.22, *β* = 99.2%). Individual analysis of predictable versus unpredictable conditions revealed significantly enhanced connectivity between the regions of IPL and PAC only for the predictable (*d* = 0.91, *β* = 90.7%) but not the unpredictable case. The significant instances appear at the same time interval as they do for the pooled conditions. No significant differences were found in the case of the averaged source signal analysis.

In connection to PAC, positive dPTE values in Figure 6(c) indicate connectivity generally directed from IPL to PAC. For looming sounds, we found a short instance of increased IPL to PAC directed connectivity after stimulus switch (*d* = 0.79, *β* = 81.4%). Again, no significant differences were observed when analyzing averaged source signals.

**Figure 7:**
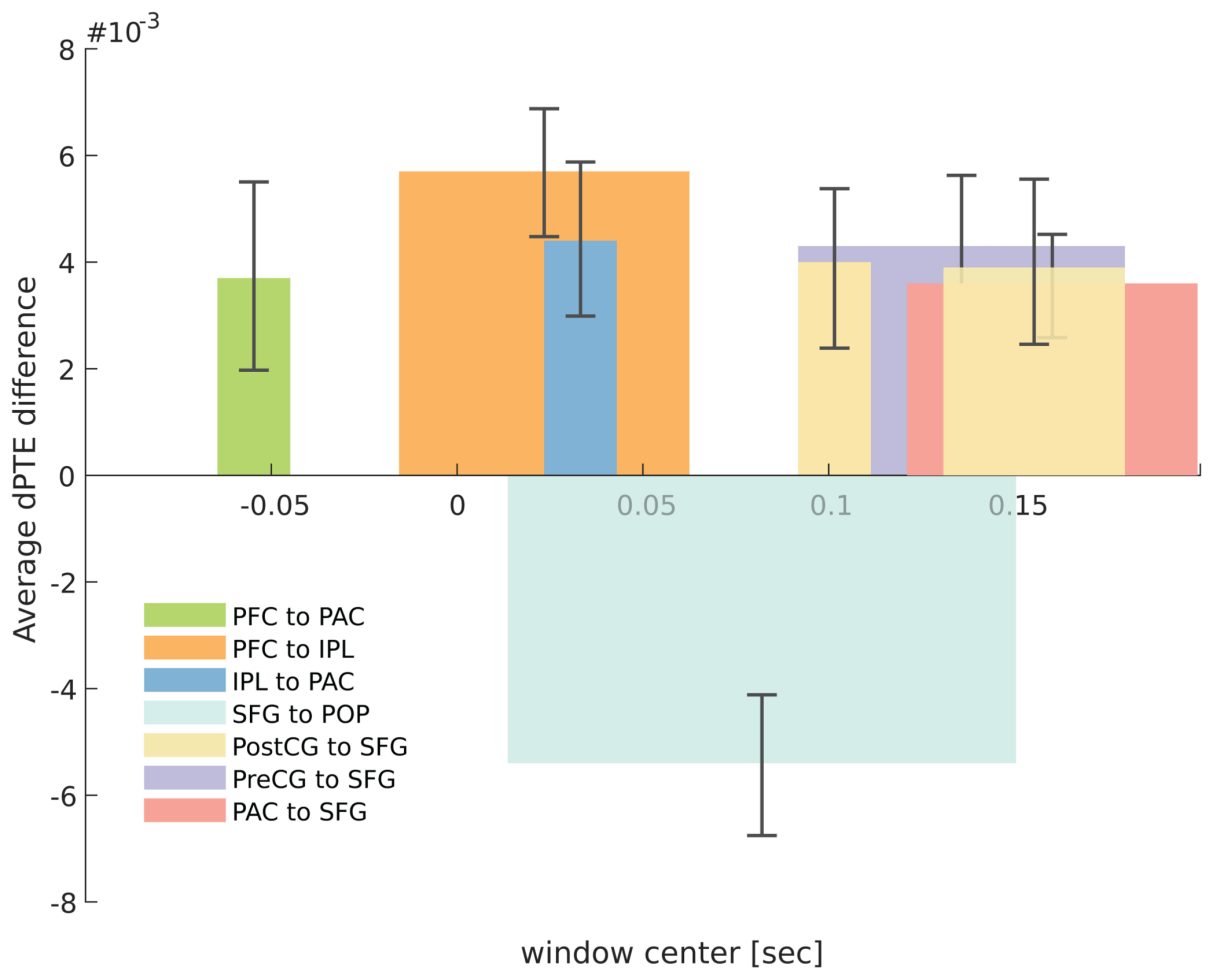
Average dPTE connectivity values over the significant occurrences of the looming bias, calculated as the difference between dPTE for looming and dPTE for receding stimuli, based on the single-trial source activities and plotted against the center of the sliding time window (duration 250 ms). Additionally to the connections presented above (Figure 6(a,b,c)) significant connections are found from PAC, PostCG and PreCG to SFG. These additional significant occurrences of the looming bias are apparent at times corresponding to the timing of maximum looming bias occurrence (120–200 ms), as indicated by Baumgartner et al. (2017). Positive dPTE values resent connectivity in the direction mention as the legend of each bar (PAC to SFG, PreCG to SFG, PostCG to SFG, SFG to POP, PFC to PAC, PFC to IPL and IPL to PAC), negative values in the opposite direction.

For all the other ROIs whose activity differed significantly in the time interval from 120 to 200 ms, single-trial analyses revealed significant instances of the looming bias in connections targeting SFG from PAC (*d* = 0.95, *β* = 92.6%), PostCG (*d* = 0.67, *β* = 67.5%) and PreCG (*d* = 0.84, *β* = 85.3%), as well as in the connection from SFG to POP (*d* = 1.06, *β* = 96.9%). The timings and strengths of those connections are summarized in Figure 7 together with the results obtained for PAC, PFC, and IPL.

## Discussion

We here explored the connectivity between temporal, parietal and frontal ROIs, based on previously collected data used to show that auditory looming bias can be elicited with constant-intensity, spectrally varying sounds (Baumgartner et al., 2017). We found that those constant-intensity looms bias cortico-cortical connectivity among different areas considered: Connectivity among the PAC, IPL and the PFC seems to take place around stimulus presentation, while interplay between the SFG and the regions of the PAC, PostCG, PreCG and POP happens at a later time consistent with previous ERP biases (120–200 ms; Baumgartner et al., 2017). Our results indicate early top-down and later bottom-up coupling induced in favor of looming sound motion.

Congruent to a recent study that promoted a top-down fronto-temporal modulation favoring the processing of looming sounds (Bidelman & Myers, 2020), our findings support the hypothesized top-down cortical connectivity between PFC and PAC. Top-down connectivity of smaller amplitude is found for the receding stimuli as well, in the same direction (PFC to PAC). However, power calculations assign a power of only 50.4% to this emergence and the timing of the connection between the areas of PFC and PAC (Figure 6(a)) seems to be rather preceding the motion onset, than persisting through it. We believe that this effect might be connected to predictive action or expectation on the side of the subjects, regarding the upcoming stimuli, rather than evoked by them. Concordantly, the bias in PFC to PAC connectivity did not persist in individual analysis of predictable versus unpredictable conditions.

By comparing predictable moving trials with static trials (which break the prediction of changes to occur), it would be possible to marginalize the components that may stem from the ability to identify the sounds or anything else that is intrinsic to the first sound. Unfortunately, the original study (Baumgartner et al., 2017) was not designed for a comparison between static and moving trials. The static trials were only introduced as catch-trials in order to measure participants' attention. For the moving trials, every combination of spectral contrast changes (looming, receding, full step, half step) was presented 120 times. Even though there were 120 static trials, the design held only 40 trials per spectral contrast (C = 0, C = 0.5, C = 1), which is too few for connectivity comparisons. In an ongoing follow-up study with high-density EEG and individualized brain anatomies, we chose a design with a balanced number of static and moving trials to allow such comparisons.

The fronto-parietal connectivity between PFC and IPL showed an interesting differentiation: looms evoked projections directed from PFC to IPL, while recedes evoked projections rather in the opposite direction (IPL to PFC; Figure 6(b)). PFC and IPL are part of the fronto-parietal network (FPN) supporting cognitive control and decision-making processes (Marek & Dosenbach, 2018; Vincent, Kahn, Snyder, Raichle, & Buckner, 2008). It is critical for our ability to coordinate behavior in a rapid, accurate, and flexible goal-driven manner, and it is anatomically positioned to integrate information from the dorsal attention and hippocampal-cortical memory systems (Singh-Curry & Husain, 2009; Vincent et al., 2008). IPL, in particular, plays an important role in maintaining attentive control on current task goals and accumulating evidence in a probability learning task, on the one hand, as well as responding to salient new information or alerting stimuli in the environment, on the other (Singh-Curry & Husain, 2009). Hence, the two directions of connectivity between PFC and IPL that we observed may potentially reflect those two roles. While looming sounds may trigger alerts projected from PFC to IPL, receding sounds may only reflect the maintenance of attentive control and accumulation of probabilistic evidence directed from IPL to PFC.

In addition to the IPL-PFC projection, we found post-switch connectivity directed from IPL to PAC to be enhanced for looming sounds (Figure 6(c)). The enhanced information flow from the IPL may mediate the function of enhanced object identification in the case of looming sounds (Singh-Curry & Husain, 2009) via the dorsal auditory pathway. Since the connectivity bias only persisted in predictable but not in unpredictable cases, predictability of the stimulus seems to play a role in enhancing feedback projections.

The observed involvement of parietal regions in the preferential processing of looming stimuli is consistent with previous fMRI results (Bach et al., 2008; Seifritz et al., 2002). Parietal regions include the association cortices that support auditory spatial processing as well as higher level object recognition and classification for further stimulus analysis, as part of the dorsal auditory network (Bizley & Cohen, 2013). Around the timing of interest (120–200 ms), all significant connections are linked with SFG. Since it is a frontal area, this may be connected to enhanced attention orientation for the more salient stimulus (involving both feedback and feed-forward connections), consistent with the saliency of looming stimuli. The SFG is also involved in preparatory processes for response selection and execution (i.e., premotor cortex connectivity) as well as enhanced decision-making processes (Rushworth, Walton, Kennerley, & Bannerman, 2004), all functions essential for an automatic reaction to a threatening stimulus. Our results therefore extend the previous knowledge by indicating how those regions may be connected.

Significant results were only obtained on the basis of single-trial analyses. Since time-locked averaging removes desynchronized activity from the output signal, results of the trial averages are considered to reflect only synchronized activity, commonly evoked by bottom-up projections. Conversely, analyses based on single trials may also contain correlates of desynchronized activity, commonly induced by top-down projections (Makeig, Debener, Onton, & Delorme, 2004). The lack of effects in average-trial-based analyses might either be methodologically based (dPTE reflects mainly induced brain processes; Mäkinen, Tiitinen, & May, 2005) or neurally based (specific processing of looming sounds rather mediated by induced functional connections).

Although there are more established and frequently used methods to assess network connectivity (e.g., Dynamic Causal Modeling; DCM), we deliberately followed a functional connectivity approach: the current data were initially collected for an ERP study; as such, they pose limitations on source localization accuracy (32 electrodes; non-individualized anatomies and electrode positions). For that reason, we chose to follow a functional connectivity approach, allowing us to draw modest conclusions regarding source correlations first, before moving toward stronger and better grounded conclusions through our ongoing follow-up study (high-density EEG; individualized electrode locations and MRI scans), where the data quality will allow for applying more state-of-the-art methodology. The choice of PTE as the connectivity measure largely relies on our target to test previous connectivity findings for complementary stimuli. In the process of its implementation we came across several questions regarding its application (time windows, trial configuration, delay estimates, etc.). We have investigated most of these aspects, but are still slightly reserved regarding our results. We believe there are more appropriate methods for drawing causal conclusions regarding connectivity, which we are aiming to employ in our upcoming studies.

For the purpose of the present study we re-analyzed archived data, previously collected for a study designed to assess ERPs in channel space. Having used a 32-channel scalp EEG with no individualized anatomies and electrode positions, the source localization accuracy is restricted. As such, there might be a higher amount of noise introduced within each ROI. Especially in the case of the PAC, a very narrow region in the temporal lobe, the attributed results might actually be originating more broadly from neighboring areas on STG.

In conclusion, our results seem to confirm that top-down projections originating from frontal decision-making areas are biased in favor of looming sounds and target not only sensory areas in the temporal lobe, as demonstrated earlier (Bidelman & Myers, 2020), but also parietal areas associated with processing of spatial and emotional information (Engelen, Zhan, Sack, & de Gelder, 2018). Yet, these top-down directed biases occurred at time points slightly preceding or happening around motion onset and thus are considered to reflect anticipatory processes. At time points following the motion onset, biases were found in the exact opposite direction. In an ongoing follow-up study with optimized design, we are aiming to solve discrepancies in reaction time data on looming bias (see Bach et al., 2015, 2009, 2008), broaden our knowledge on spectrally evoked looms, and investigate predictability effects. With this high-density EEG study (featuring individual brain anatomies and electrode positions) we are aiming to better understand the networks underlying the looming bias, as well as how they are formed, developed and maintained.
